# The Role of Fixed-Dose Desmopressin in Hemostatic Outcomes of Native and Transplant Kidney Biopsies in a Tertiary Referral Hospital

**DOI:** 10.3390/healthcare13131553

**Published:** 2025-06-29

**Authors:** Nisrin Bifari, Yasser Alatawi, Wesam S. Abdel-Razaq, Mohammad S. Shawaqfeh, Abdulkareem M. Albekairy, Fayez Hejaili, Ghassan F. Shattat, Mohammed Alkathiri, Yousef A. Alrajhi, Khalid A. Al Sulaiman, Abdulmalik M. Alkatheri

**Affiliations:** 1 Pharmacy Practices Department, College of Pharmacy, Umm Al-Qura University, Makkah 21955, Saudi Arabia; 2 Department of Pharmacy Practice, Faculty of Pharmacy, University of Tabuk, Tabuk 47512, Saudi Arabia; 3 College of Pharmacy, King Saud bin Abdulaziz University for Health Sciences, Ministry of National Guard Health Affairs, Riyadh 14611, Saudi Arabia; 4 King Abdullah International Medical Research Centre, Ministry of National Guard Health Affairs, Riyadh 11481, Saudi Arabia; 5 Pharmaceutical Care Services, King Abdulaziz Medical City, Ministry of National Guard Health Affairs, Riyadh 11426, Saudi Arabia; 6 College of Medicine, King Saud bin Abdulaziz University for Health Sciences, Ministry of National Guard Health Affairs, Riyadh 14611, Saudi Arabia; 7 College of Science and Health Professions, King Saud bin Abdulaziz University for Health Sciences, Ministry of National Guard Health Affairs, Riyadh 14611, Saudi Arabia

**Keywords:** desmopressin, kidney biopsy, hemostasis, kidney transplant, safety

## Abstract

**Introduction/Objectives**: Although desmopressin is commonly used to reduce bleeding hazards in patients undergoing kidney biopsies, its effectiveness varies among individuals. This study aims to assess the impact of desmopressin on bleeding risk and hemodynamic stability in patients undergoing kidney biopsies while also identifying potential risk factors influencing these outcomes. **Methods**: A retrospective study was conducted at King Abdulaziz Medical City to evaluate adult patients who underwent either native or transplant kidney biopsies. The collected data included demographics, comorbidities, demographics, desmopressin usage, vital signs, lab results, and bleeding events. Bleeding was defined as a composite outcome encompassing both minor and major bleeding. **Results**: Data from 210 patients who received desmopressin during kidney biopsies were analyzed alongside 200 control patients. The distribution of gender and age was comparable between the two groups. However, the types of biopsies differed significantly, with a greater number of native kidney biopsies in the desmopressin group. Desmopressin was associated with a reduced incidence of major bleeding and shorter hospital stays. Longitudinal analyses revealed significant time-dependent changes in mean arterial pressure, hemoglobin, and hematocrit, although no treatment effect was observed. Logistic regression showed no significant impact of desmopressin on composite bleeding, hypotension, or hyponatremia, though comorbidities and transplant biopsies were associated with a reduced risk of hyponatremia. **Conclusions:** Desmopressin was associated with fewer episodes of major bleeding and shorter hospital stays but had no direct effect on hemodynamic parameters. Nevertheless, further research is necessary to explore its long-term clinical impact.

## 1. Introduction

Kidney biopsy is a crucial procedure in nephrology for diagnosing and predicting outcomes in kidney diseases that carries a risk of bleeding complications [1], such as major bleeding. In terms of management, major bleeding after a percutaneous renal biopsy, can usually be managed with arterial embolization of the injured renal vessel [2]. Since Iversen and Brun’s initial description in 1951 [3], the development of kidney biopsy techniques has focused on minimizing these bleeding risks. Despite advancements, post-biopsy hematoma, hematuria, kidney loss, and even death remain recognized potential bleeding complications of kidney biopsies [4,5,6]. The risk of major bleeding is variable in most patients but indeed low. A risk score can be helpful to guide the decision concerning kidney biopsy and the choice of the procedure in kidney recipients [7]. Noteworthy is that compromised renal function patients (low eGFR), especially below 30 mL/min, are associated with increased bleeding risk in transplant kidney biopsies [8].

Desmopressin (DDAVP) is commonly used to reduce the risk of bleeding in patients undergoing kidney biopsies [9]. The available data are inadequate to support the use of DDAVP routinely prior to percutaneous kidney biopsies [10]; yet its effectiveness varies depending on various patient factors [11]. In renal transplantation, the risk factors for developing bleeding due to biopsy were younger patients and patients with a lower mean arterial pressure (MAP) and Resistive Index (RI) ≥0.8 [12]. Several studies have indicated that patients receiving a weight-based dose of desmopressin (0.3 mcg/kg) experienced a lower incidence of hematoma compared to control groups for patients at high risk for bleeding complications, particularly those with impaired renal function [13,14]. Even in an RCT, intranasal desmopressin administration effectively reduces post-biopsy kidney peri-renal hematoma formation at 24 h post-procedure [15]. Despite these encouraging outcomes, some studies have indicated that desmopressin is not recommended for routine use in low-risk individuals due to the potential risks of hyponatremia that may outweigh the benefits in this population [16,17]. In addition, prebiopsy intranasal desmopressin use was associated with a significant reduction in overall bleeding complications, including major and minor complications, and there was no reduction in the rate of other major complications and interventions [18].

Research on the effectiveness of desmopressin in preventing bleeding complications following a kidney biopsy has produced mixed results, indicating a need for further investigation to clarify the role of desmopressin in this context. This study evaluated the therapeutic outcome of using a fixed dose of desmopressin to prevent bleeding following a native or transplant kidney biopsy. The study also examined the incidence of hyponatremia and hypotension following desmopressin administration and explored the risk factors contributing to these adverse events.

## 2. Materials and Methods

### 2.1. Study Design

A single-center, retrospective observational cohort study was conducted on patients who underwent native or transplant kidney biopsies at King Abdulaziz Medical City, a tertiary healthcare governmental hospital in Riyadh, Saudi Arabia. The study included adult patients aged 18 years or older who were admitted to the hospital between 1 January 2016 and 30 June 2022. Data from eligible patients were collected, including demographics, comorbidities, and the procedure type (i.e., native or transplant kidney biopsy).

Patients were given a fixed dose of 20.0 micrograms of desmopressin to reduce the risk of bleeding during the kidney biopsy, regardless of their body weight. The optimal time to administer desmopressin before a kidney biopsy is 30 min to 2 h prior to the procedure. In all our patients, as per protocol, desmopressin was given via the intravenous route.

To analyze desmopressin use, patients were stratified based on their risk of bleeding complications following the kidney biopsy. High-risk patients include those with pre-existing coagulation disorders, renal impairment, anticoagulant or antiplatelet medication use, uncontrolled hypertension, and advanced age with comorbidities that necessitate careful assessment to minimize hemorrhagic risks [1]. In contrast, low-risk patients did not exhibit these factors. Additional information gathered included vital signs (pre- and post-desmopressin for two days afterward), laboratory data, and bleeding events. Chronic kidney disease was categorized based on the estimated glomerular filtration rate (eGFR), and acute kidney injury (AKI) was diagnosed according to KDIGO guidelines. The eGFR is calculated using the re-expressed 4-variable MDRD equation (using standardized creatinine). Blood pressure measurements at the time of the kidney biopsy procedure were also recorded. Bleeding events were defined as a composite of hemoglobin decline, blood transfusion, hematuria, and symptomatic hematoma. Exclusion criteria included patients with active bleeding within 72 h before the biopsy, those already using intranasal or oral desmopressin, and individuals with severe hyponatremia (sodium level < 125 mmol/L).

Major bleeding includes one or more of the following events: it requires blood transfusion, causes significant hemoglobin to drop, requires intervention to stop bleeding, leads to hemodynamic instability, and causes prolonged hospitalization or ICU admission. Minor bleeding includes one of the following: transient, self-limiting macroscopic hematuria resolving within 24–48 h, microscopic hematuria, small, and asymptomatic perinephric hematoma that resolves spontaneously and does not require transfusion or intervention to stop bleeding.

The kidney biopsy protocol in all patients was an ultrasound-guided procedure. In addition, as per the protocol, all biopsies were aimed at cortical tissue. Furthermore, the standardized protocol of two biopsy cores was obtained for each patient, and all biopsies were performed by or under the direct supervision of experienced nephrologists/interventional radiologists.

### 2.2. Data Management and Analysis

Continuous variables were assessed for normality and described as mean, standard deviation, median, and interquartile range. Categorical variables were expressed as frequencies and proportions. As appropriate, differences between the desmopressin group and those who did not receive desmopressin were evaluated using the chi-square test or the unpaired t-test. The longitudinal changes in various measured parameters were analyzed using two-way repeated measures ANOVA analysis or a mixed model for datasets with missing values. Risk factors for bleeding events as a composite outcome, hyponatremia, and hypotension were analyzed using multivariable logistic regression models across desmopressin groups, providing corresponding odds ratios (ORs) and 95% confidence intervals (CIs). Statistical analyses were performed using the GraphPad Prism^®^ statistical software package version 9.0 (San Diego, CA, USA), and a *p*-value of less than 0.05 designates statistical significance.

All research activities will be conducted according to relevant ethical guidelines and approval provided by the Institutional Review Board at King Abdullah International Medical Research Center, Riyadh, Saudi Arabia (reference number NRC22R/410/09). Patient confidentiality was maintained throughout the study.

## 3. Results

Data was retrieved from the hospital’s electronic medical records for 210 patients who underwent either native or transplant kidney biopsies and received desmopressin to mitigate the risk of bleeding. A comparable sample of 204 adult patients was included, which served as the control group that underwent the same procedure without receiving desmopressin. However, four patients were subsequently excluded from the control group due to incomplete records. The general characteristics of the remaining 410 patients are detailed in Table 1. Gender distribution was similar between the two groups, with males comprising the majority at 60.0% in the desmopressin group and 63.5% in the control group (*p* = 0.466). The mean age was comparable, at 42.3 ± 14.7 years (range 18–83) in the desmopressin group and 42.9 ± 15.9 years (range 18–85) in the control group (*p* = 0.685). Age distribution across categories also showed no statistically significant differences between the two groups (*p* = 0.741), indicating that age and gender were well-balanced between the two cohorts. This suggests that any observed effects on the study outcomes are unlikely to be confounded by these variables.

Interestingly, a statistically significant difference was realized in the type of biopsy procedure between the two groups (*p* < 0.001), with a higher proportion of native kidney biopsies in the desmopressin group (74.8% vs. 58.5%) and more transplant biopsies in the non-desmopressin group (41.5% vs. 25.2%). This imbalance may influence bleeding risk and other clinical outcomes.

The foremost clinically significant differences observed in the study were major bleeding, which was significantly lower in the desmopressin group (4.8% vs. 10.0%, *p* = 0.042), and the length of hospital stay, which was also significantly shorter in the desmopressin group (median 7 vs. 10 days, *p* = 0.009). No other baseline characteristics showed statistically significant differences between the two groups.

Table 2 presents a comparative analysis of desmopressin use in kidney biopsy patients, stratified by bleeding risk. The study included 210 patients, with 187 classified as high-risk and twenty-three as low-risk patients. Among the studied variables, age was the only statistically significant factor (*p* = 0.008), with high-risk patients averaging 43.2 ± 14.9 years compared to 34.6 ± 10.3 years in the low-risk group. Other variables, including gender distribution, procedure type, timing of administration, dosage received, and dosage groups, showed no statistically significant differences. Composite bleeding rates were also similar between high-risk and low-risk groups (p = 0.744), suggesting no significant association between bleeding outcomes and risk stratification.

**Figure 1:**
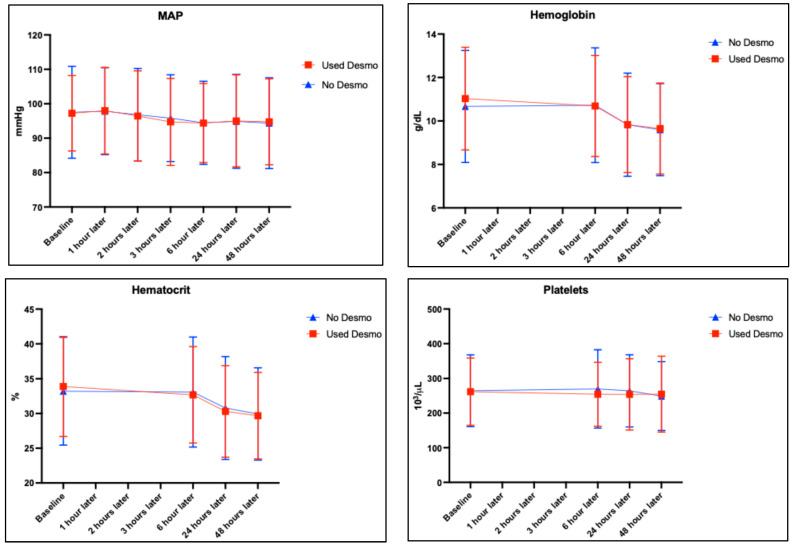
Longitudinal changes in mean arterial pressure (MAP), hemoglobin, hematocrit, and platelet levels over time in patients treated with desmopressin (red line) and those not given desmopressin (blue line). Statistical significances were determined using two-way repeated measures ANOVA analysis or a mixed model for datasets with missing values.

Figure 1 shows the longitudinal changes in the mean arterial pressure (MAP), hemoglobin (HGB), hematocrit (HTC), and platelet levels over time in the two patient groups. MAP analysis revealed a statistically significant reduction across time intervals in both groups (*p* < 0.001), but no significant difference was observed between treatments (*p* = 0.895). Together, the hemoglobin and hematocrit analyses indicate a statistically significant decline across time intervals for both groups (*p* < 0.001) and a significant interaction between time and treatments (*p* < 0.001) but not between treatments alone (*p* = 0.767 and 0.757, respectively). Platelet count analysis showed no statistically significant differences across time or between treatments (*p* = 0.393 and *p* = 0.265, respectively).

Finally. Table 3 displays the logistic regression analysis, which assessed the impact of various risk factors on bleeding events, hypotension, and hyponatremia. Desmopressin use was not significantly associated with bleeding (OR = 1.06, *p* = 0.826), hypotension (OR = 0.83, *p* = 0.574), or hyponatremia (OR = 0.99, *p* = 0.952), indicating no strong protective or harmful effect. Peculiarly, comorbidities and higher CCI scores were associated with a lower risk of hyponatremia (OR = 0.26, *p* = 0.015 and OR = 0.56, *p* = 0.025, respectively). While transplant kidney biopsies did not significantly affect bleeding or hypotension risk, they were associated with a reduced hyponatremia risk (OR = 0.57, *p* = 0.026). Other factors, including gender, age, BMI, and antithrombotic use, were not significant predictors for any outcome. Overall, the analysis suggests that desmopressin does not significantly alter bleeding or other hemodynamic risks.

## 4. Discussion

Desmopressin is commonly utilized for bleeding prophylaxis and to minimize transfusion requirements in various clinical settings, including adult cardiac surgery [19], urgent invasive procedures for patients on antithrombotic agents [20], and kidney biopsies in high-risk individuals [14]. A group suggested a standard use of desmopressin for bleeding prophylaxis prior to kidney biopsy. This protocol included measurements of serum sodium levels, along with ultrasound and hemoglobin as part of the patient monitoring. However, this proposed protocol was not adequately evaluated in a clinical practice [21]. Furthermore, desmopressin plays a key role in managing congenital bleeding disorders [20,21,22].

A study highlighted a significant association of the female sex with both minor and major bleeding complications. There was no sound hypothesis to support this risk factor; however, the increased bleeding risk in females may be due to their smaller kidney size, resulting in the biopsy needle penetrating deeper into the renal parenchyma [23]. However, the use of desmopressin in kidney biopsies is still a topic of ongoing discussion and research. Conflicting evidence exists regarding the efficacy of desmopressin in preventing bleeding complications following a kidney biopsy, such as hematoma formation or the need for blood transfusions. While some studies suggest a clear benefit for high-risk patients, others find no significant reduction in post-biopsy bleeding complications [24,25] or even indicate an increased bleeding risk after kidney biopsies in low-risk individuals [26,27]. The heterogeneity in patient populations and the variability in desmopressin administration protocols across different studies contribute to these conflicting findings.

The kidney biopsy protocol in all patients was an ultrasound-guided procedure. The justification for its use included the advantage of real-time visualization of the needle’s path as it enters the kidney and the avoidance of ionizing radiation associated with CT-guided biopsies. In addition, some studies suggested a lower overall complication rate with US-guided biopsies in comparison with CT-guided biopsies. It is also known that CT-guided biopsies have some advantages that make them an alternative option in special and more complicated cases; however, in our study, all the procedures were of one type, managed by expert evaluators. In addition, as per the protocol, all biopsies aimed for cortical tissue to ensure adequate diagnostic yield and minimize medullary complications. Furthermore, the standardized protocol of two biopsy cores was obtained for each patient. Regarding the performers’ experience, all biopsies were performed by or under the direct supervision of experienced nephrologists/interventional radiologists with adequate years of experience in performing kidney biopsies.

A common approach to desmopressin dosing as a hemostatic agent is to use a weight-based dose, typically at 0.3 mcg/kg, administered subcutaneously [28,29] or intravenously [30]. Although desmopressin is also available in an intranasal dosage form, which is easier to administer, parenteral administration allows for more precise dosing and faster onset. Desmopressin is often diluted in saline and administered 15–30 min before the biopsy [31]. This dosing strategy tailors the drug effect to the individual patient’s physiological needs. Another study did not find any utility of prophylactic desmopressin use before the kidney biopsy in patients with impaired renal function [32].

While weight-based dosing is prevalent, some clinical practices employ a fixed-dose approach, particularly with intranasal administration at a dose of 300 µg in adults and 150 µg in children [33]. Nonetheless, patients with impaired kidney function may require dose adjustments due to altered drug clearance. It is also important to keep in mind that the fixed-dose regimen of desmopressin may result in under- or overdosing due to variations in patient body mass, making it a less standardized approach in kidney biopsies. Specifically, the consistency and predictability of bleeding risk may be less dependable compared to the other approach.

Due to the scarcity of studies using a strictly fixed desmopressin dosage for kidney biopsies, this study aims to evaluate the efficacy of a fixed 20.0 microgram (µg) dose of desmopressin in preventing bleeding following native or transplant kidney biopsies. The use of such an unconventional low fixed dose was inspired by two recent studies. First, Franquiz et al. (2018) [33] compared the efficacy of two different weight-based desmopressin dosing strategies in bleeding control. They reported no significant difference between patients who received intravenous weight-adjusted desmopressin doses and those receiving standard weight-based dosing for hemostatic indications after surgical procedures [33]. Second, Furqan, Sham et al. (2020) demonstrated a relatively safe yet effective outcome for a half-dose of desmopressin at 0.15 mcg/kg to achieve hemostasis in patients who underwent a variety of endoscopic and biopsy procedures [34]. While weight-based dosing is the precise dosing in the treatment of hemophilia A and von Willebrand’s syndrome (e.g., 0.3 mcg/kg IV), the fixed dose is often used for biopsy prophylaxis for several reasons. First, the response is predictable for patients with mild to moderate coagulation defects or uremia and thehe fixed dose is usually adequate. Second, for practical benefit, astandardized fixed dose with a known concentration makes it an acceptable option for timely intervention and distances the clinician from complex calculations.

The results of the present study provide a comparison of desmopressin use in kidney biopsy patients, categorized by their risk of bleeding complications. The analysis showed that age was the only factor significantly different between the two groups, with high-risk patients being older on average compared to the low-risk group. This may suggest that age is a notable differentiating factor for using desmopressin. Remarkably, desmopressin was an effective intervention associated with significantly reduced major bleeding and shorter hospital stays, but it did not significantly alter minor bleeding events. Furthermore, desmopressin at the fixed dose used appears to be safe since it was not associated with a significant impact on hemodynamic parameters or electrolyte imbalance, indicating no strong protective or harmful effects. However, other potential adverse events associated with desmopressin, such as hyponatremia and thromboembolic events, should always be carefully considered against the potential benefits. The proposed mechanism of action of desmopressin in preventing bleeding after the biopsy is primarily due to the release of the von Willebrand factor from endothelial cells, which helps platelet adhesion to injured vessels and increases factor VIII levels [35].

In a subgroup of patients with impaired renal function, desmopressin administration prior to a renal biopsy does not reduce the frequency of bleeding complications or the post-biopsy perinephric hematoma volume [36]. The fact that desmopressin reduced major bleeding but not the composite suggests that it is more effective at preventing the serious and clinically significant bleeding complications, and this is a highly desirable outcome. However, the lack of effect on the composite outcome suggests that desmopressin may not be as effective in preventing minor and self-limiting bleeding events. This might be explained by a few hypotheses as follows. a. Even in the presence of desmopressin, some degree of minor bleeding may occur due to the invasive nature of the biopsy procedure (physiological limit). b. It is known that minor bleeding is more likely to be detected in post-biopsy imaging, even if it is clinically relevant. c. Desmopressin may raise the clotting factor levels sufficiently to prevent large, uncontrolled bleeding, but not enough to completely stop all minor bleeding.

Finally, further well-designed, adequately powered research is still warranted to definitively determine the role of desmopressin in reducing overall bleeding in kidney biopsy patients, particularly across different patient subgroups, while also investigating its long-term effects on hemodynamic stability and blood parameters. Until more robust evidence is available, clinical decisions regarding desmopressin administration should be individualized, considering patient-specific risk factors and a thorough discussion of the potential risks.

The study has some limitations related to the retrospective study design with a limited sample size. This may affect the power of the study and make the results more prone to outliers, confounders, and recall bias. However, this study can generate hypotheses and provide preliminary data towards the practice of using fixed-dose desmopressin in preventing biopsy-related bleeding.

## 5. Conclusions

The study results suggest that desmopressin use was associated with a lower incidence of major bleeding and a shorter hospital stay. Longitudinal analysis revealed significant changes in mean arterial pressure, hemoglobin, and hematocrit levels over time, but there was no direct effect of desmopressin on these parameters. Nonetheless, the optimal dosing strategy for desmopressin continues to be a subject of debate, and individual patient factors must be considered. Until more robust evidence is available, clinical decisions regarding desmopressin administration should be individualized, considering patient-specific risk factors and a thorough discussion of the potential risks.

## Figures and Tables

**Table 1:** General characteristics of involved patients who underwent native or transplant kidney biopsies; n = 410.

Variable	Desmopressin Used (n = 210)	No Desmopressin (n = 200)	*p*-Value ^†^
Gender, n (%) Male Female	126 (60.0%) 84 (40.0%)	127 (63.5%) 73 (36.5%)	0.466
Age (in years) Mean ± (SD) Median (range, IQR)	42.3 ± (14.7) 40.0 (18–83, 24.8)	42.9 ± (15.9) 42.0 (18–85, 26.0)	0.685
Age (in years), n (%) Young adults: 18–34 Middle-aged adults: 35–49 Older adults: 50–64 Elderly: ≥ 65	72 (34.3%) 64 (30.5%) 57 (27.1%) 17 (8.1%)	75 (37.5%) 52 (26.0%) 54 (27.0%) 19 (9.5%)	0.741
BMI, n (%) <18.5 (underweight) 18.5–24.9 (healthy weight) 25–29.9 (overweight) ≥30 (obesity)	6 (2.9%) 40 (19.0%) 66 (31.4%) 98 (46.7%)	6 (3.0%) 51 (25.5%) 57 (28.5%) 86 (53.0%)	0.470
Comorbidities, n (%) ^‡^ None Hypertension CKD Diabetes mellitus Carcinomas Liver disease Others ^¶^	30 (14.3%) 114 (54.3%) 159 (75.7%) 70 (33.3%) 8 (3.8%) 8 (3.8%) 51 (24.3%)	31 (15.5%) 110 (55.0%) 146 (73.0%) 65 (32.5%) 8 (4.0%) 6 (3.0%) 40 (20%)	0.730 0.885 0.529 0.858 0.921 0.652 -
CCI scores, n (%) Mild (1–2) Moderate (3–4) Severe (≥5)	127 (60.5%) 63 (30.0%) 20 (9.5%)	131 (65.5%) 56 (28.0%) 13 (6.5%)	0.424
Medication history, n (%) ^‡^ None Antiplatelets Anticoagulants Antiplatelets and anticoagulants Steroids	86 (41.0%) 20 (9.5%) 59 (28.1%) 2 (1.0%) 81 (38.6%)	77 (38.5%) 24 (12.0%) 61 (30.5%) 5 (2.5%) 88 (44.0%)	0.612 0.418 0.593 0.227 0.264
Antithrombotic therapy continued through biopsy, n (%) ^#^ Antiplatelets Anticoagulants	9 (45.9%) 38 (64.4%)	10 (41.7%) 40 (65.6%)	0.811 0.824
Type of procedure, n (%) Naive kidney biopsy Transplant kidney biopsy	157 (74.8%) 53 (25.2%)	117 (58.5%) 83 (41.5%)	<0.001
Preprocedural MAP (in mmHg) Mean ± (SD) Median (range, IQR)	97.3 ± (11.0) 97.7 (61–122, 15.2)	97.5 ± (13.4) 97.8 (62–137, 18.5)	0.821
Preprocedural HTN, n (%) No Yes	119 (56.7%) 91 (43.3%)	109 (54.5%) 91 (45.5%)	0.659
Pre-BUN (mg/dL) Mean ± (SD) Median (range, IQR)	16.2 ± (10.2) 14.2 (2.0–68, 12.8)	15.0 ± (9.9) 12.0 (2.5–54, 13.6)	0.239
Pre-ALT (U/L) Mean ± (SD) Median (range, IQR)	18.1 ± (10.0) 15.0 (5.0–47, 15.0)	22.0 ± (17.1) 16.5 (5–125, 15.8)	0.055
Pre-AST (U/L) Mean ± (SD) Median (range, IQR)	19.3 ± (9.2) 17 (5.0–54, 8.0)	21.2 ± (13.6) 17.0 (6.0–93, 9.8)	0.241
Pre-sodium (U/L) Mean ± (SD) Median (range, IQR)	135.7 ± (4.1) 137 (121–146, 6.0)	135.1 ± (4.4) 136 (122–148, 6.0)	0.121
eGFR, n (%) Normal (≥60 mL/min) Reduced (<60 mL/min) Missing data (n = 1 + 2)	20 (9.6%) 189 (90.4%)	23 (11.6%) 175 (88.4%)	0.502
CKD stages, n (%) ^§^ Stage 1/2 Stage 3a Stage 3b Stage 4 Stage 5 Missing data (n = 1 + 2) ^¶^	20 (9.6%) 29 (13.9%) 46 (22.0%) 45 (21.5%) 69 (33.0%) -	24 (12.1%) 31 (15.7%) 45 (22.7%) 56 (28.3%) 42 (21.2%) -	0.095
Undergoing dialysis, n (%) No Yes	143 (68.1%) 67 (31.9%)	139 (69.5%) 61 (30.5%)	0.759
AKI stages, n (%) None Stage 1 Stage 2 Stage 3	75 (35.7%) 23 (11.0%) 15 (7.1%) 97 (46.2%)	53 (26.5%) 29 (14.5%) 18 (9.0%) 100 (50.0%)	0.208
Blood parameters, ^Ψ^ n (%) Low hemoglobin Low hematocrit Low platelet count Missing data (n = 2 + 2) ^¶^	139 (66.5%) 155 (74.2%) 13 (6.3%) -	146 (73.7%) 153 (77.3%) 15 (7.6%) -	0.112 0.465 0.598 -
Minor bleeding, n (%) No Yes	180 (85.7%) 30 (14.3%)	177 (88.5%) 23 (11.5%)	0.401
Major bleeding, n (%) No Yes	200 (95.2%) 10 (4.8%)	180 (90.0%) 20 (10.0%)	0.042
Composite bleeding, ^Φ^ n (%) No Yes	174 (82.9%) 36 (17.1%)	167 (83.5%) 33 (16.5%)	0.862
Need for blood transfusion, n (%) No Yes	200 (95.2%) 10 (4.8%)	181 (90.5%) 19 (9.5%)	0.061
Length of hospital stay (in days) Mean ± (SD) Median (range, IQR)	10.7 ± (12.6) 7 (1–99, 11.0)	18.4 ± (40.3) 10 (1–371, 15.0)	0.009
Length of hospital stay (in days) <3 3–10 >10 Missing data (n = 1) ^¶^	45 (21.4%) 92 (43.8%) 73 (34.8%)	41 (20.6%) 58 (29.1%) 100 (50.3%)	0.003

^†^ Calculated using the chi-square/Fisher exact test for categorical variables or the unpaired t-test for continuous variables. ^‡^ Percentages are calculated from the total number of patients in the respective group. ^¶^ Other, unknown, or missing data were excluded from the statistical calculations. ^#^ Percentages are calculated from the total number of antithrombotic users in the respective group. ^§^ Percentages are calculated from the total number of CKD patients in the respective group. ^Ψ^ Low hemoglobin levels are defined as less than 13.0 g/dL for men and 11.0 g/dL for women; low hematocrit levels are below 41% for men and 36% for women; and low platelet count is considered when the platelet count falls below 140,000 platelets per microliter of circulating blood. ^Φ^ Composite bleeding refers to a combination of major bleeding with a hemoglobin decrease of greater than 3 g/dL that necessitates intervention and minor bleeding where the overt blood flow is minimal and does not meet major bleeding criteria. Abbreviations: BMI (Body Mass Index); CCI (Charlson Comorbidity Index); MAP (mean arterial pressure); HTN (hypertension); BUN (Blood Urea Nitrogen); ALT (Alanine Transaminase); AST (Aspartate Aminotransferase); eGFR (estimated glomerular filtration rate); CKD (chronic kidney disease); AKI (acute kidney injury).

**Table 2:** Details of desmopressin use in kidney biopsy patients stratified by risk for bleeding complications; n = 210.

Variable	All (n = 210)	High Risk ^†^ (n = 187)	Low Risk (n = 23)	*p*-Value ^‡^
Gender, n (%) Male Female	126 (60.0%) 84 (40.0%)	113 (60.4%) 74 (39.6%)	13 (56.5%) 10 (43.5%)	0.718
Age (in years) Mean ± (SD) Median (range, IQR)	42.3 ± (14.7) 40.0 (18–83, 24.8)	43.2 ± (14.9) 43.0 (18–83, 23.0)	34.6 ± (10.3) 36.0 (19–58, 13.5)	0.008
Type of procedure, n (%) Naive kidney biopsy Transplant kidney biopsy	157 (74.8%) 53 (25.2%)	137 (73.3%) 50 (26.7%)	20 (87.0%) 3 (13.0%)	0.241
Time of administration, n (%) Pre-procedure Post-procedure Missing data (n = 4)	197 (95.6%) 9 (4.4%)	178 (96.7%) 6 (3.3%)	19 (86.4%) 3 (13.6%)	0.089
Dose received (in mcg/kg) Mean ± (SD) Median (range, IQR)	0.26 ± (0.07) 0.25 (0.14–0.53, 0.09)	0.26 ± (0.07) 0.25 (0.14–0.53, 0.09)	0.26 ± (0.07) 0.24 (0.16–0.42, 0.12)	0.573
Dosage groups, n (%) Adequate (0.3–0.4 mcg/kg) Underdosed (<0.3 mcg/kg) Overdosed (>0.4 mcg/kg)	50 (23.8%) 153 (72.9%) 7 (3.3%)	44 (23.5%) 137 (73.3%) 6 (3.2%)	6 (26.1%) 16 (69.6%) 1 (4.3%)	0.916
Composite bleeding ^Φ^ n (%) No Yes	174 (82.9%) 36 (17.1%)	156 (83.4%) 31 (16.6%)	18 (78.3%) 5 (21.7%)	0.744

^†^ High-risk patients include those with pre-existing bleeding disorders, renal impairment, anticoagulant or antiplatelet medication use, uncontrolled hypertension, and advanced age with comorbidities. ^‡^ Calculated between high-risk and low-risk patients using an appropriate statistical test. ^Φ^ Composite bleeding refers to a combination of major bleeding with a hemoglobin decrease of greater than 3 g/dL that necessitates intervention and minor bleeding where the overt blood flow is minimal and does not meet major bleeding criteria.

**Table 3:** Logistic regression analysis of risk factors associated with the study outcomes.

Variable	Bleeding Events ^†^		Hypotension		Hyponatremia	
	Odds Ratio (95% CI)	*p*-Value	Odds Ratio (95% CI)	*p*-Value	Odds Ratio (95% CI)	*p*-Value
Desmopressin used No **Yes**	1.06 (0.62 to 1.82)	0.826	0.83 (0.42 to 1.61)	0.574	0.99 (0.62 to 1.57)	0.952
Gender Male **Female**	0.87 (0.51 to 1.51)	0.624	0.61 (0.32 to 1.19)	0.147	1.32 (0.82 to 2.16)	0.259
Age group Less than 50 years **50 years and older**	1.44 (0.80 to 2.67)	0.229	0.94 (0.46 to 1.97)	0.877	0.79 (0.48 to 1.31)	0.362
BMI <25 under and healthy **≥25 over and obese**	0.99 (0.53 to 1.80)	0.981	1.38 (0.64 to 2.81)	0.388	0.96 (0.56 to 1.61)	0.876
Comorbidity None **Yes**	1.10 (0.50 to 2.33)	0.801	1.41 (0.50 to 3.62)	0.493	0.26 (0.08 to 0.70)	0.015
CCI scores Mild (1–2) **Moderate/severe (≥3)**	0.92 (0.50 to 1.73)	0.800	0.74 (0.34 to 1.61)	0.454	0.56 (0.34 to 0.93)	0.025
Antithrombotic drugs No **Yes**	1.09 (0.61 to 1.96)	0.776	0.84 (0.42 to 1.73)	0.635	0.83 (0.51 to 1.37)	0.468
Type of biopsy Naive kidney **Transplant kidney**	1.75 (0.94 to 3.39)	0.085	1.27 (0.60 to 2.85)	0.551	0.57 (0.35 to 0.94)	0.026
Bleeding events No **Yes**	—	—	1.16 (0.47 to 2.55)	0.731	1.39 (0.75 to 2.52)	0.283
Hypotension events No **Yes**	0.86 (0.39 to 2.10)	0.719	—	—	0.76 (0.36 to 1.63)	0.460
Hyponatremia events No **Yes**	0.71 (0.39 to 1.31)	0.264	0.75 (0.36 to 1.60)	0.440	—	—

† Defined as a composite bleeding, which combines both major and minor bleeding. **Bolded** group compared to the non-bolded reference.

## Data Availability

The original contributions presented in this study are included in the article. Further inquiries can be directed to the corresponding author.
